# Mindfulness and Self-compassion as Unique and Common Predictors of Affect in the General Population

**DOI:** 10.1007/s12671-016-0568-y

**Published:** 2016-07-08

**Authors:** Angélica López, Robbert Sanderman, Maya J. Schroevers

**Affiliations:** 1 Department of Health Psychology, University of Groningen, University Medical Center Groningen, Hanzeplein 1, De Brug FA12, Groningen, 9700 RB The Netherlands; 2 Department of Psychology, Health and Technology, University of Twente, Enschede, The Netherlands

**Keywords:** Mindfulness, Self-compassion, Depressive symptoms, Negative affect, Positive affect

## Abstract

In contrast to the increased research interest in the benefits of mindfulness and self-compassion, relatively few studies have examined their unique and combined effects in predicting affect. This cross-sectional study examined the predictive value of mindfulness and self-compassion for depressive symptoms, negative affect, and positive affect in a large representative sample of community adults (*N* = 1736). The Five Facets of Mindfulness Questionnaire (FFMQ) was used as a measure of mindfulness and the Self-Compassion Scale (SCS) as a measure of self-compassion. Five FFMQ facets were explored: observe, describe, act with awareness, non-judgment, and non-reactivity. Two SCS facets were explored: its positive items (SCS Pos) and its negative items (SCS Neg). When simultaneously examining all seven facets of mindfulness and self-compassion, three of the five FFMQ facets and SCS Neg significantly predicted both depressive symptoms and negative affect, with SCS Neg and act with awareness being the strongest predictors. These findings suggest that a harsh attitude towards oneself and a lack of attention when acting have the greatest value in predicting the presence of psychological symptoms. With respect to positive affect, four of the five FFMQ facets (except non-judgment) were significant predictors, with no unique predictive value of the two SCS’s facets, suggesting that mindfulness is a more important predictor of positive affect than self-compassion, as measured by the FFMQ and SCS.

## Introduction

During the last decades, the western world has seen a flourishing of interest in the practice of mindfulness with an increased number of studies investigating its benefits and those of mindfulness-based interventions (Fjorback et al. 2011). Mindfulness, a concept rooted in Buddhist mediation traditions, involves being aware of the present experience, by paying attention on purpose and without judgment (Kabat-Zinn 1994). Mindfulness has been operationalized in different ways, sometimes as a single-factor construct and others as a multifaceted construct. Empirical reviews have shown that mindfulness has beneficial effects on psychological health, such as reduced psychological symptoms and increased subjective wellbeing (Keng et al. 2011; Khoury et al. 2013).

More recently, psychologists have also moved their attention towards the study of self-compassion, a construct closely related to mindfulness. Self-compassion, also rooted in Buddhist traditions, is commonly defined as treating oneself with kindness and understanding when facing suffering, seeing one’s failures as part of the human condition, and having a balanced awareness of painful thoughts and emotions (Neff 2003a). Self-compassion has also proven to be beneficial for psychological health, with a meta-analysis showing that higher levels of self-compassion are associated with lower psychological symptoms (MacBeth and Gumley 2012).

Conceptually, mindfulness and self-compassion are strongly linked constructs that go hand on hand (Maex 2011; Neff 2003a). For instance, it has been argued that a mindful attention should be characterized by a kind, non-judgmental attitude (Segal et al. 2002), and that in turn, to hold compassion towards oneself, it is necessary to be mindfully aware of the ongoing experience (Neff 2003a). Yet, the focus of mindfulness and self-compassion differs. Whereas, mindfulness refers to a non-judgmental awareness of a wide range of present-moment experiences (e.g., emotions, cognitions, bodily sensations, sounds, etc.), whether pleasant, neutral or unpleasant, self-compassion involves the awareness and response to suffering of the self, thus focussing more on emotion regulation (Boellinghaus et al. 2014).

One of the most commonly used questionnaires to measure mindfulness is the Five Facets of Mindfulness Questionnaire (FFMQ, Baer et al. 2006). This questionnaire measures five mindfulness skills, i.e., observe, describe, act with awareness, non-reactivity, and non-judgment, that can be combined into a total score of mindfulness. Although this total score has been often used, recent research suggests that it is more valid to look at the separate five facets (Williams et al. 2014). Regarding self-compassion, the vast majority of research has used the Self-Compassion Scale (SCS) as it is currently the only scale available to measure self-compassion (Neff 2003b). The SCS contains six subscales, three of them measure a compassionate approach to suffering (i.e., self-kindness, common humanity, and mindfulness) and the other three measure the opposite, a harsh and critical attitude towards oneself (i.e., self-judgment, isolation, and over-identification). Researchers have usually examined these six subscales as well as a total score of self-compassion. However, recent evidence indicated that it is not meaningful to use a total score, since a six-factor hierarchical structure has not being replicated in community, clinical, nor meditating samples (Williams et al. 2014). Some studies have found evidence for a six-factor correlated structured for the SCS (Petrocchi et al. 2013; Hupfeld and Ruffieux 2011), whereas others showed support for a two-factor structure with one factor containing the positive items and the other the negative items (Costa et al. 2015; López et al. 2015). Recent evidence showed that these two factors correlate differently to psychological symptoms (Muris and Petrocchi 2016); in line, some authors recommend the exclusive use of the positive items as a measure of self-compassion (Muris 2015; Muris et al. 2016).

So far, the empirical evidence for the separate and combined effects of mindfulness and self-compassion for psychological wellbeing is limited to a handful of studies. In a sample of patients with mixed anxiety and depression interested in a self-help intervention program, Van Dam et al. (2011) found that a total SCS score of self-compassion was a more powerful predictor of psychological symptom severity and quality of life, than a single-factor score of mindfulness (as measured by the Mindful Attention Awareness Scale, MAAS; Brown and Ryan 2003). Unfortunately, as a result of using the MAAS, the study could not examine the different facets of mindfulness. Similar results were found in a combined sample of highly educated meditators and non-meditators, when using the total score of a multi-facet measure of mindfulness (i.e., Five Facets of Mindfulness Questionnaire; FFMQ; Baer et al. 2006) and a total score of self-compassion (SCS) (Baer et al. 2012). However, when looking at a subscale level, mindfulness and self-compassion facets predicted a similar amount of variance of psychological wellbeing. It should be noted that in these analyses, several subscales of the FFMQ and SCS were excluded and others were combined, making it difficult to draw definite conclusions. In a relatively small sample of undergraduate students, Woodruff et al. (2013) also found that when using total scores, self-compassion explained a larger amount of variance of depressive symptoms and negative affect than mindfulness, yet for positive affect they explained about similar amount of variance. When including all 11 facets of mindfulness and self-compassion, few facets were significant unique predictors. A similar result was found in a combined sample of undergraduate students and demographically comparable community members (Hollis-Walker and Colosimo 2011).

The aim of the present study was to examine the unique predictive value of mindfulness and self-compassion, as measured by the FFMQ and the SCS, for affect. In order to overcome limitations of previous studies (i.e., selective samples including students, highly educated people, or anxious and depressed individuals interested in psychological help), we conducted this study in a large representative sample from the general population with equivalent gender distribution and a broad age range, allowing greater generalizability of the findings. In addition, we used multi-facet measures of both constructs to allow the examination of the distinct facets of mindfulness and self-compassion. Finally, as previous literature has shown differential associations of mindfulness and self-compassion with negative and positive indicators of psychological functioning, we focused on both negative and positive indicators of affect (Schroevers and Brandsma 2010).

## Method

### Participants

A total of 1736 adults constituted the study sample, of whom 54.7 % was female. The mean age was 54.9 years old (SD = 16.8), ranging from 20 to 96 years old. The majority of the participants completed middle education (48.4 %), followed by high (31.5 %) and low (20 %) education. A 76.3 % of the participants was married or cohabitating, 9.6 % was single, 7.1 % was widowed, 4.1 % was divorced, and 2.9 % others.

### Procedure

The community-based sample was selected from the register offices of five middle-size cities in the Netherlands. The characteristics of the sample were representative of the Dutch population in age and gender distributions. Participants were sent a letter explaining the aim of the study, an informed consent and the self-report questionnaire package. Informed consent was obtained from all participants. Participants that failed to complete 15 % or more of the questionnaire package were excluded.

### Measures

Mindfulness was assessed with the Five Facets of Mindfulness Questionnaire (FFMQ; Baer et al. 2006; De Bruin et al. 2012). This 39-item scale contains five facets: observe, describe, act with awareness, non-judgment, and non-reactivity. Participants rated in a five-point likert scale the degree to which every statement was true for them ranging from 1 (*never or very rarely true*) to 5 (*very often or always true*). A total score for each facet can be calculated after reversing the negative worded items and summing the totality of the items. Higher scores are indicative of greater levels of mindfulness. In this study, the internal consistency was good for describe (α = 0.87), act with awareness (α = 0.84), and non-judgment (α = 0.85), and acceptable for non-reactivity (α = 0.74) and observe (α = 0.76).

Self-compassion was measured with the Self-Compassion Scale (SCS; Neff and Vonk 2009; Neff 2003b). Originally, the SCS is divided into six subscales: self-kindness, self-judgment, common humanity, isolation, mindfulness, and over-identification. The items can be rated on a five-point likert scale ranging from 1 (*almost never*) to 5 (*almost always*). After reversing the negative-worded items, a total score can be calculated with higher scores indicating greater self-compassion. The SCS total score can range from 24 to 120. In this study, the internal consistency was good for the SCS total score (α = 0.86).

*Depressive symptoms* were assessed with the Center of Epidemiologic Studies Depression Scale (CES-D; Bouma et al. 1995; Radloff 1977; Schroevers et al. 2000). The CES-D is a 20-item self-report instrument designed to measure current levels of depressive symptomatology in the general population. Total scores may range from 0 to 60, with higher scores indicating more depressive symptoms. In this study, the CES-D showed a good internal consistency (α = 0.89). *Positive and negative affect* were measured with the 20-item Positive and Negative Affect Schedule (PANAS; Peeters et al. 1999; Watson et al. 1988). This instrument is divided into two 10-item scales that assess feelings of activeness, enthusiasm and alertness (i.e., positive affect), and subjective distress and unpleasant engagement (i.e., negative affect). The internal consistency was good for positive affect (α = 0.88) as well as for negative affect (α = 0.87).

### Data Analyses

Prior to conducting the main analyses, the factor structures of the FFMQ and SCS were tested with confirmatory factor analysis using weighted least squares method based on polychoric correlation matrix, in MPlus, version 7.1, and with exploratory factor analysis (EFA) in SPSS 20.0 using maximum likelihood method with varimax rotation. The main analyses were conducted in SPSS 20.0. Pearson’s correlations between all study variables were computed to examine their interrelationships. Next, multiple regressions were conducted to examine the predictive value of mindfulness and self-compassion for depressive symptoms, negative affect, and positive affect. The FFMQ (i.e., observe, describe, act with awareness, non-reactivity, and non-judgment) and SCS (i.e., SCS positive items and SCS negative items) facets were entered simultaneously as predictor variables with separate regressions for the three indicators of affect.

## Results

Following Baer et al. (2006), a five-factor structure was tested for the FFMQ with CFA using both items and parcels. Results showed that a five-factor correlated model did not fit well, neither when using items (*χ*2/df = 15.25, CFI = 0.778, TLI = 0.762, RMSEA = 0.091, WRMR = 4.035) nor when using parcels (*χ*2/df = 19.33, CFI = 0.816, TLI = 0.759, RMSEA = 0.103, SMRS = 0.092). Similarly, a five-factor hierarchical model did not fit good the data for items (*χ*2/df = 20.93, CFI = 0.688, TLI = 0.668, RMSEA = 0.107, WRMR = 5.291) and did not converge when using parcels. Consequently, we performed EFA. The scree plot indicated the presence of five factors. A second EFA was conducted with five fixed factors. After rotation, all items load on the original five factors, with a total explained variance of 49 %.

Following Neff (2003b), a six-factor hierarchical model for the SCS was tested with CFA but it could not be estimated due to weak correlations between some pairs of the six factors that prevented a higher order factor from emerging. Subsequently, a model with six correlated factors was tested but also showed an insufficient fit (*χ*2/df = 16.19, CFI = 0.898, TLI = 0.881, RMSEA = 0.094, WRMR = 3.171). EFA was performed, with the scree plot indicating the presence of two factors. Thus, a second EFA was conducted with two fixed factors. After rotation, the 12 negatively formulated items loaded on one factor and the 12 positively formulated items loaded on the other factor. The total explained variance of this two-factor solution was 45.4 %. Such a two-factor model has also been found by others (Costa et al. 2015).

Based on these results, this study focused on the separate five facets of the FFMQ and the two facets of the SCS, one containing its positive items/subscales (referred to as SCS Pos) and the other containing its negative items/subscales (referred to as SCS Neg). That is, the SCS’s positive subscales (i.e., self-kindness, common humanity, and mindfulness) were combined into SCS Pos and the SCS’s negative subscales (i.e., self-judgment, isolation and over-identification) were combined into SCS Neg.

First, we examined the interrelationships among the facets of self-compassion and mindfulness. The SCS Pos was significantly, and most strongly related to the mindfulness facet of non-reactivity (*r* = 0.51), and to a lesser extent, but still moderately, to observe (*r* = 0.37) and describe (*r* = 0.35). In contrast, SCS Neg was significantly and most strongly related to act with awareness (*r* = −0.55) and non-judgment (*r* = −0.59).

With respect to affect, depressive symptoms and negative affect were significantly and moderately to strongly related to the mindfulness facets of act with awareness (*r* = −0.50 and *r* = −0.47, respectively) and non-judgment (*r* = −0.43 and *r* = −0.45, respectively), and to SCS Neg (*r* = 0.52 and *r* = 0.53, respectively). In contrast, positive affect was significantly and most strongly related to describe (*r* = 0.35) and non-reactivity (*r* = 0.31), and to a lesser extent, to observe (*r* = 28) and SCS Pos (*r* = 0.29) (Table 1).

**Table 1:** Means, standard deviations, and inter-correlations between study variables

		Mean	SD	1	2	3	4	5	6	7	8	9
1	SCS Pos	36.24	7.98	–								
2	SCS Neg	28.18	9.18	−0.11^***^	–							
3	FFMQ-obs	24.98	5.56	0.37^***^	0.09^***^	–						
4	FFMQ-des	27.24	5.75	0.35^***^	−0.21^***^	0.28^***^	–					
5	FFMQ-act	28.88	5.14	0.15^***^	−0.55^***^	−0.05	0.30^***^	–				
6	FFMQ-nonj	29.07	5.68	0.08	−0.59^***^	−0.23^***^	0.13^***^	0.50^***^	–			
7	FFMQ-nonr	21.61	4.40	0.51^***^	−0.08	0.41^***^	0.35^***^	0.09^***^	−0.05	–		
8	CES-D	9.45	8.30	−0.25^***^	0.52^***^	−0.01	–0.22^***^	−0.50^***^	−0.43^***^	−0.20^***^	–	
9	PANAS-NA	15.72	5.90	−0.15^***^	0.53^***^	0.07	−0.18^***^	−0.47^***^	−0.45^***^	−0.15^***^	0.68^***^	–
10	PANAS-PA	30.14	7.37	0.29^***^	−0.11^***^	0.28^***^	0.35^***^	0.22^***^	0.09^***^	0.31^***^	−0.36^***^	−0.11^***^

*SCS Pos* Self-compassion Scale’s positive items, *SCS Neg* Self-compassion Scale’s negative items, *FFMQ-obs* observe, *FFMQ-des* describe, *FFMQ-act* act with awareness, *FFMQ-nonj* non-judgment, *FFMQ-nonr* non-reactivity, *CES-D* Center of Epidemiologic Studies Depression Scale, *PANAS-NA* Positive and Negative Affect Schedule-Negative affect scale, *PANAS-PA* Positive and Negative Affect Schedule-Positive affect scale

^***^ *p* < 0.001

The combination of FFMQ’s facets, SCS Pos and SCS Neg significantly predicted depressive symptoms (*F* (7, 1695) = 147.87, *p* < 0.001) and negative affect (*F* (7, 1696) = 133.10, *p* < 0.001), explaining 37.7 and 35.2 % of the outcomes’ variance, respectively (Table 2). The combination of facets of the FFMQ, SCS Pos, and SCS Neg also significantly predicted positive affect (*F* (7, 1693) = 62.31, *p* < 0.001), explaining 20.2 % of the outcome’s variance (Table 2).

**Table 2:** Prediction of outcome variables by FFMQ’s facets, SCS Pos, and SCS Neg

	β	*sr* ^2^	Common variance	*R* ^2^
Depressive symptoms				
SCS Pos	−0.126^***^	0.011	0.269	0.377
SCS Neg	0.278^***^	0.042		
FFMQ-obs	0.019	0.000		
FFMQ-des	0.005	0.000		
FFMQ-act	−0.252^***^	0.039		
FFMQ-nonj	−0.133^***^	0.010		
FFMQ-nonr	−0.108^***^	0.008		
Negative affect				
SCS Pos	−0.037	0.001	0.250	0.352
SCS Neg	0.299^***^	0.049		
FFMQ-obs	0.053	0.002		
FFMQ-des	0.010	0.002		
FFMQ-act	−0.214^***^	0.028		
FFMQ-nonj	−0.156^***^	0.014		
FFMQ-nonr	−0.121^***^	0.009		
Positive affect				
SCS Pos	0.075	0.004	0.125	0.202
SCS Neg	0.056	0.002		
FFMQ-obs	0.164^***^	0.020		
FFMQ-des	0.187^***^	0.027		
FFMQ-act	0.141^***^	0.012		
FFMQ-nonj	0.071	0.003		
FFMQ-nonr	0.133^***^	0.012		

*R* ^2^ *=* percentage of variance explained by predictors; *sr* ^2^ = semi-partial correlation squared (i.e., percentage of variance explained by one specific predictor above and beyond all others); common variance = percentage of predicted variance redundant between the predictors

*SCS Pos* Self-compassion Scale’s positive items, *SCS Neg* Self-compassion Scale’s negative items, *FFMQ-obs* observe, *FFMQ-des* describe, *FFMQ-act* act with awareness, *FFMQ-nonj* non-judgment, *FFMQ-nonr* non-reactivity

^***^ *p* < 0.001

Three of the five FFMQ’s facets, as well as SCS Pos and SCS Neg, significantly predicted depressive symptoms, with SCS Neg and act with awareness being the strongest predictors. Results were similar for negative affect, with the only difference that the predictive value of SCS Pos was not significant for negative affect. With respect to positive affect, four of the five FFMQ facets were significant predictors (i.e., observe, describe, act with awareness, and non-reactivity), with no unique predictive value of the two SCS’s facets.

Of the total explained variance for depressive symptoms, the FFMQ’s facets uniquely predicted 15 %, compared to 14 % uniquely predicted by the two SCS’s facets, with 71 % being common variance. Similarly, of the total explained variance for negative affect, the FFMQ’s facets uniquely predicted 16 %, compared to 14 % uniquely predicted by the two SCS’s facets, with 70 % being common variance. In contrast, of the total explained variance for positive affect, the FFMQ’s facets uniquely predicted 36 %, compared to only 3 % uniquely predicted by the two SCS’s facets, and 61 % being common variance.

## Discussion

This study examined the unique and common predictive value of mindfulness and self-compassion (as measured by the FFMQ and SCS) for affect, in a large representative sample from the general population. We focused on the five facets of the FFMQ and two facets of the SCS, one containing its positive items/subscales (referred to as SCS Pos, including the original subscales of self-kindness, common humanity, and mindfulness) and the other containing its negative items/subscales (referred to as SCS Neg, including the original subscales of self-judgment, isolation, and over-identification). Results indicate that the combination of the FFMQ’s facets, SCS Pos and SCS Neg, explained a larger amount of variance for depressive symptoms and negative affect, compared to positive affect. The FFMQ’s facets, SCS Pos and SCS Neg, uniquely predicted a similar amount of variance of depressive symptoms and negative affect (with around 70 % common variance). In contrast, of the total explained variance for positive affect, the FFMQ’s facets uniquely predicted 36 %, compared to only 3 % uniquely predicted by the SCS Pos and SCS Neg (with 61 % common variance). For depressive symptoms, three of the five FFMQ’s facets, the SCS Neg and the SCS Pos were significant predictors, with SCS Neg and act with awareness being the strongest predictors. Results were similar for negative affect with the only difference that SCS Pos was not a significant predictor. With respect to positive affect, four of the five FFMQ’s facets were significant predictors, with no unique predictive value of the SCS Pos and SCS Neg.

A key finding is that the FFMQ’s and SCS’s facets predicted a similar amount of unique variance for depressive symptoms and negative affect. This finding is consistent with previous studies, indicating that mindfulness and self-compassion predict an equal amount of variance of psychological symptoms when examining their facets (instead of using their total scores) (Woodruff et al. 2013). Previous evidence using total scores generally found that the SCS was a stronger predictor of negative affect compared to the FFMQ (Woodruff et al. 2013). Altogether, ours and previous studies’ results suggest that the FFMQ loses predictive value for negative indicators of affect when it is consolidated into a total score (Baer et al. 2006; Woodruff et al. 2013).

Regarding positive affect, our main finding is that the FFMQ’s facets predicted a larger percentage of unique variance than the two SCS’s facets. A previous study did not find a significant prediction of the FFMQ’s and SCS’s facets on positive affect (Woodruff et al. 2013). When looking at the simple correlations, we did find a significant, marginally moderate, relationship between SCS Pos and positive affect. A possible explanation for why this relationship was not found in the regression analyses is that the predictive value of SCS Pos was covered to a large extent by the FFMQ facets. Another explanation can be the broader focus of mindfulness compared to self-compassion. Mindfulness skills can be applied to any ongoing experience, while self-compassion is restricted to experiences that involve suffering (Baer et al. 2012; Boellinghaus et al. 2014). The FFMQ assesses a set of mindfulness skills that, when present, can produce a shift in individuals’ perceptions, giving more clarity and objectivity to moment-by-moment experience (Shapiro et al. 2006). In turn, this can facilitate cognitive, behavioral, and emotional flexibility, positively influencing individuals’ wellbeing (Shapiro et al. 2006).

When scoping into the specific facets, act with awareness showed to be a strong predictor, accounting for the largest amount of unique variance across the outcomes, particularly for the negative indicators of affect. This is in line with previous studies (Hollis-Walker and Colosimo 2011; Woodruff et al. 2013) and suggests that being mindfully aware of the ongoing experience is an important contributor of affect. One of the ways in which being aware is associated with affect might be through disengaging individuals from harmful automatic thoughts and unhealthy behaviors (Brown and Ryan 2003), which in turn facilitates the self-regulation of behavior to fulfill one’s needs and values, and subsequently a better mood (Ryan and Deci 2008).

Additionally, and in line with the findings of Woodruff et al. (2013), though to a lesser extent, non-reactivity also showed to be a significant predictor of all outcome measures, suggesting that not reacting impulsively to ongoing experiences, particularly to thoughts and emotions, has important benefits for affect. In addition, the SCS Neg showed to be the strongest predictor of psychological symptoms, indicating that a harsh attitude towards oneself has important negative implications for wellbeing. Non-judgment also showed to significantly predict psychological symptoms. These two facets measure a self-critical attitude that has shown to be an important determinant for depressive symptoms (Dunkley et al. 2006 2009). Finally, our findings suggest that the mindfulness skills to be observing, able to describe internal and external experiences, non-reacting to emotions and thoughts, and to act with awareness, are all predictors of positive affect. This is in line with previous literature, showing that specifically changes in being aware and observing of experiences significantly predicted changes in positive affect (Schroevers and Brandsma 2010). More research, both correlational as well as intervention studies, is needed to examine closely the distinct relationships between the facets of mindfulness and self-compassion with negative and positive indicators of wellbeing and mediators explaining these relationships.

It is worth to notice that the SCS’s negative items showed to be a stronger predictor of negative affect than the SCS’s positive items. This finding suggests that the predictive value of the SCS total score over the FFMQ total score, as found in previous studies (Woodruff et al. 2013), could be mainly related to the predictive value of these negative items. In line with this, Van Dam et al. (2011) found that among the SCS’s subscales, self-judgment and isolation (in our study combined) were the strongest predictors of symptom severity. Others have also found strong associations between the SCS’s negative items and depressive symptoms, more so than the ones of the SCS’s positive items (Mills et al. 2007; Wasylkiw et al. 2012). It has been suggested that the SCS’s negative items seem to be measuring self-coldness instead of self-compassion (López et al. 2015; Gilbert et al. 2011). In the light of our results, this might indicate that mainly self-coldness, and not self-compassion, is an important predictor of psychological symptoms.

Our findings have some clinical implications. Mindfulness and self-compassion showed to have unique contributions on affect, suggesting that both are important for individuals’ wellbeing. Particularly, a range of mindfulness skills seem to have a greater benefit on positive affect, thus playing an important protective role, whereas a lack of self-compassion in terms of self-coldness (i.e., SCS Neg) seems to have a notorious negative effect, predicting more strongly psychological symptoms in combination with a lack of awareness for doing daily activities. The latter suggests that to improve psychological symptoms, it is important to work on individuals’ harsh attitudes towards themselves as well as stimulate a mindful awareness and reduction of functioning on automatic pilot.

Some limitations need to be considered when interpreting our findings. In this study, we compared the predictive value of two commonly used measures of mindfulness and self-compassion; however, our results are constrained to the characteristics and limitations of these questionnaires. Some parts of the SCS and FFMQ overlap in content, which hinders the interpretation of their unique effects on affect. In particular, the SCS’s items related to self-judgment overlap in content with the FFMQ’s non-judgment items. Also, the SCS’s items related to a mindful approach to difficult experiences and not being over-identified by such experiences overlap in content with the FFMQ’s non-reactivity items. Another limitation is the cross-sectional design, which precludes us from drawing conclusions about the direction of the relationships. For instance, we cannot conclude whether higher levels of mindfulness and self-compassion lead to lower negative affect and more positive affect, or whether this is the other way around. Previous longitudinal research has shown evidence for the first hypothesis, with higher level of mindfulness leading to lower levels of psychological symptoms (Snippe et al. 2015). More longitudinal studies are necessary to confirm our findings.

It is also important to notice that we focused on a representative sample from the general population; thus, our sample is not representative for participants of mindfulness- or self-compassion-based interventions, who generally experience higher levels of psychological symptoms. In addition, the prior mediation experience of our sample was unknown, yet mean levels of mindfulness were comparable to levels found in other samples from the general population. Given these characteristics of our sample, it is important that future research examines the unique benefits of mindfulness and self-compassion in samples differing in the severity of psychological symptoms and in meditation experience. It should also be noted that the current study was performed among Dutch individuals, with the few previous studies on this topic being conducted primarily in samples from the USA and Canada. Given this low number of studies on the unique and common predictive value of mindfulness and self-compassion for psychological wellbeing, and the great differences between previous samples (e.g., students, persons with anxiety and need for help) and our sample, it is difficult to conclude to what extent results may have been affected by the Dutch culture. Cross-cultural research is needed to examine possible cultural differences in the predictive value of mindfulness and self-compassion for the experience of negative and positive affect.

Overall, our findings point out the importance of distinguishing the different facets of mindfulness and self-compassion when examining their benefits for psychological functioning, rather than using the total scores of multi-facet self-report questionnaires. Mindfulness and self-compassion, as measured by the FFMQ and SCS, were found to be equally important in predicting levels of psychological symptoms, with mindfulness also being related to the experience of positive affect. It is important to note that a lack of self-compassion in terms of self-coldness was found to be a stronger predictor of psychological symptoms than self-compassion in terms of self-kindness. This suggests that it is important to find ways by which individuals can reduce these harmful self-related thoughts and feelings.
